# General Immune Status and Oral Microbiology in Patients with Different Forms of Periodontitis and Healthy Control Subjects

**DOI:** 10.1371/journal.pone.0109187

**Published:** 2014-10-09

**Authors:** Jana Schmidt, Holger Jentsch, Catalina-Suzana Stingu, Ulrich Sack

**Affiliations:** 1 Institute for Clinical Immunology, University of Leipzig, Leipzig, Germany; Translational Centre for Regenerative Medicine (TRM), University of Leipzig, Leipzig, Germany; 2 Department of Cariology, Endodontology and Periodontology, University of Leipzig, Leipzig, Germany; 3 Institute for Medical Microbiology and Epidemiology of Infectious Diseases, University of Leipzig, Leipzig, Germany; University of Florida, United States of America

## Abstract

**Objective:**

Immunological processes in the etiopathogenesis of periodontitis, especially the aggressive form, are not well understood. This study examined clinical as well as systemic immunological and local microbiological features in healthy controls and patients with different forms of periodontitis.

**Materials and Methods:**

14 healthy subjects, 15 patients diagnosed with aggressive periodontitis, and 11 patients with chronic periodontitis were recruited. Periodontal examination was performed and peripheral blood was collected from each patient. Lymphocyte populations as well as the release of cytokines by T-helper cells were determined by flow cytometry and enzyme linked immunosorbent spot assay. Subgingival plaque samples were taken from each individual and immediately cultivated for microbiological examination.

**Results:**

When stimulating peripheral blood mononuclear cells (PBMCs) with lipopolysaccharide, a higher IL-1β release was found in patients with moderate chronic periodontitis compared to the other groups (p<0.01). Numbers of B-cells, naïve and transitional B-cells, memory B-cells, and switched memory B-cells were within the reference range for all groups, but patients with chronic periodontitis showed the highest percentage of memory B-cells without class switch (p = 0.01). The subgingival plaque differed quantitatively as well as qualitatively with a higher number of Gram-negative anaerobic species in periodontitis patients. *Prevotella denticola* was found more often in patients with aggressive periodontitis (p<0.001) but did not show an association to any of the systemic immunological findings. *Porphyromonas gingivalis*, which was only found in patients with moderate chronic periodontitis, seems to be associated with an activation of the systemic immune response.

**Conclusion:**

Differences between aggressive periodontitis and moderate chronic periodontitis are evident, which raises the question of an inadequate balance between systemic immune response and bacterial infection in aggressive periodontitis.

## Introduction

Periodontitis is an inflammatory disease affected by a variety of factors. Smoking and age as well as diabetes mellitus, which seems to be in a bidirectional relationship with periodontitis [1], and stress are known risk factors. Furthermore, gender [2], education level [3], and immunological diseases such as HIV-infection [4] influence the presentation of periodontal diseases. On the other hand, periodontal inflammation seems to promote cardiovascular diseases, stroke, and pneumonia [5]–[7]. Therefore, especially severe forms of periodontitis are of common and interdisciplinary interest. Chronic periodontitis is a quite common disease in adult patients characterized by pocket formation and/or recession [8]. While progressive loss of periodontal attachment occurs slowly to moderately [9], local risk factors, e.g. bacterial plaque and calculus, accelerate loss of attachment particularly rapidly [10].

Aggressive periodontitis is characterized by its severe and fast progressing destructive course in young individuals, which often leads to tooth loss early in life followed by the need for prosthetic treatment. Family aggregation is described [11]. Discrepancies between local factors and disease severity are often presented [10]. Diagnosis should be based upon all available information referring to general and special anamnesis and after careful clinical examination of a patient [12].

The microbiological differences between the chronic and aggressive forms of periodontitis are a topic of extensive debate. It is generally accepted that potential pathogenic bacteria belong to the commensal oral microflora [13]. Rescala et al. could not find any differences in the microbial profile between patients with chronic and aggressive periodontitis [14]. Most species, like *Porphyromonas gingivalis*, seem to be associated with probing depth rather than with aggressive or chronic periodontitis [15]. Other authors showed associations between different pathogens (*P. gingivalis, T. forsythia, T. denticola*) and chronic periodontitis [16].

Innate and adaptive immunity are both arms of the immune system defending the organism against pathogens. The first wall of defense against invading pathogens is the innate immunity, which is activated within a few hours. If the innate immunity is not able to limit an infection, adaptive immunity develops accompanied by the development of immunological memory [17]. Lipopolysaccharide (LPS) is a potent stimulator of the immune system due to its endotoxic, highly conserved lipid region (lipid A) [18]. Different Gram-negative bacteria express different forms of LPS on their surface enabling them to activate pattern recognition receptors (PRR) as toll-like-receptors (TLR) [19]. Enterobacteria such as *Escherichia coli* stimulate TLR-4 as does the LPS found on the periodontal pathogen *Porphyromonas gingivalis* [20]. A cluster of cytokines released by peripheral blood mononuclear cells (PBMCs) when stimulated by LPS could give additional information about patients' susceptibility to periodontitis. Cytokines, such as Interleukin (IL)-1β, as well as chemokines, such as IL-8, play an important role in immune regulation, managing the maturation of dendritic cells and leading to the initiation of the transition from innate to adaptive immune response [21]. This transition is mainly initiated in regional lymph nodes by dendritic cells presenting bacterial antigens to naïve T-cells with their corresponding receptor [22]. A suitable T-cell is activated by interaction with an antigen-presenting cell which presents antigen fragments on its major histocompatibility complex (MHC). The MHC-antigen-complex activates the T-cell-receptor, thereby stimulating cytokine release by CD4 T-helper cells or activating cytotoxic T-cells which express CD8 [17]. T-helper cells express receptors, which only interact with MHC-II bound antigens. They play a major role in modulating the immune response without being phagocytic and express only partially cytotoxic activity. Different types of T-helper cells play a role in the pathogenesis of periodontal disease: Th2-cells induce the humoral B-cell response by releasing IL-4, IL-5, and IL-10 on the one hand and suppress the T-cell mediated immune response on the other hand [23]. Therefore, this Th-cell subset is associated with non-protective antibody response resulting in aggressive periodontitis and progression of periodontal lesions. Contrary to the Th2-cells, Th1-cells are associated with stable periodontal lesions [24], [25]. This can be explained by the cellular immune response stimulated by IL-12 and IFN-γ, which are characteristic Th-1 cytokines [26], and the inhibition of the differentiation of osteoclasts by self-fusion of macrophages [27]. Furthermore, the immune response mediated by Th1-cells is associated with the generation of specific antibodies [16]. Besides Th1- and Th2-cells, Th17-cells and regulatory Th-cells are described: Th17-cells produce IL-17, which induces the release of IL-6, IL-8, and prostaglandin E_2_. Furthermore, the activation of osteoclasts by Th17-cells has been shown [16], which leads to the progression of (alveolar) bone loss. Regulatory Th-cells (CD4+CD25+) were found to have anti-inflammatory and protective effects by which they possibly control gingival inflammation as well as alveolar bone resorption [28]. They were found in periodontal lesions in cases of increasing percentages of B-cells [29].

Memory B-cells express CD27, a member of the TNF-receptor-family, on their surface, which is also expressed by naïve T-cells. CD27 binds CD70, a surface protein of dendritic cells and thereby mediates the interaction between dendritic cells und B-cells [30].

As shown above, different immunological aspects are suspected to play roles in the development of periodontal diseases. Furthermore, microbiological features are reported to determine pathogenesis. In our study, we intended to examine clinical as well as immunological and microbiological features in patients with different forms of periodontitis and healthy controls. We hypothesized that there would be differences in the percentage of CD4+ T-cells producing IL-4 between patients suffering from aggressive periodontitis compared to patients with chronic periodontitis and healthy controls. The Th1/Th2 ratio was expected to be lower in patients with aggressive periodontitis compared to the other groups, because Th1-responses are linked with a potent immune response as explained above. Furthermore, we hypothesized that PBMCs' expression of IL-1β and IL-8 upon stimulation with standardized extracts of bacterial antigens would differentiate the groups. Differences were expected in the microbiological profile between healthy controls and patients, but not between the different groups of patients suffering from moderate chronic periodontitis (CP) or aggressive periodontitis (AP).

## Materials and Methods

### Ethics statement

We recruited patients for our study from the Department of Cariology, Endodontology, and Periodontology, University of Leipzig, Germany, according to the ethics committee approval of the Ethics Committee of the Faculty of Medicine, University of Leipzig, Germany (registry number 151-2009-06072009, in German). Every patient was informed about the aim and the course of the study and signed a consent form as well as a data protection policy.

### Subject selection

Every subject filled out a questionnaire about their medical history and some main aspects of oral hygiene. The subjects included were in good general health (no history of diabetes, hepatitis or HIV infection; no immunosuppressive chemotherapy; no diseases which compromise the immune system) and had at least 20 teeth. All subjects had to be of European descent and non-smokers for at least five years or occasional smokers with a cigarette consumption of up to ten cigarettes per week [31]. Patients were excluded if there was any immunosuppressive therapy in their medical history or if they took antibiotics during the previous six months. Female patients were excluded if pregnant or lactating.

The diagnosis was based on clinical and radiographic parameters and in accordance with the Classification of Periodontal Diseases [32]. We conducted a six-point-measurement of each tooth, recording probing depth (PD), clinical attachment loss (CAL), tooth mobility (TM), and bleeding on probing (BOP). To assess oral hygiene, the Interproximal Plaque Index (IPI) adapted from Lange [33] was assessed after coloring the plaque with “Mira 2 Ton”, Hager and Werken (Leimen, Germany). Periodontal measurements were performed by two dentists using the rigid Parodontometer UNC#UNC15, Hu Friedy (Leimen, Germany). The intra- and inter-examiner reproducibility of measurement was tested before commencing the study.

According to the measurement results the subjects were assigned into one of three clinical categories:

1. Healthy control group (HC): Subjects aged between 21 to 35 years with no history of periodontal disease and no PD >3 mm (excluding third molars) or CAL >1 mm.
2. Moderate chronic periodontitis (CP): Patients between 30 and 60 years old with tooth sites exhibiting 3 to 4 mm of CAL and ≥4 mm PD, in at least three teeth in two or more different quadrants. Fast progression of periodontal disease led to exclusion.
3. Aggressive periodontitis (AP): The diagnosis was made before age 35 or retrospectively based on x-ray images made before the 35^th^ birthday of the patient [34]. Rapidly progressive loss of attachment was confirmed and family aggregation likely (not absolutely necessary) in the patients. Patient tooth sites exhibited ≥5 mm PD and CAL in at least two remaining teeth. Radiographic bone loss was at least 50%, in at least two different teeth [34].

### Reagents

LPS from *Escherichia coli* (L2654) was obtained from Sigma Aldrich (Saint Louis, Missouri USA) and ultrapure LPS from *Porphyromonas gingivalis* from Invivo Gen (San Diego, USA); CWPS (Pneumococcal-cell-wall-polysaccharide mixture) was obtained from Statens Serum Institute (Copenhagen, Denmark), and CEF peptide pool from R&D Systems (Minneapolis, USA). RPMI 1640 with L-Glutamine, HEPES (N-2-hydroxyethylpiperazine-N′-2-ethanesulfonic acid) and low-endotoxin fetal calf serum (FCS) were purchased from PAA (Pasching, Austria). Pairs of antibodies against IL-1β and IL-8 suitable for enzyme-linked immunosorbent spot (ELISpot) assay were obtained from BioLegend/BIOZOL (Eching, Germany). Streptavidin alkaline phosphatase (Streptavidin-ALP) was from Mabtech (Hamburg, Germany) and BCIP/NBT (5-bromo-4-chloro-3-indolyl-phosphate/nitro blue tetrazolium) substrate was from Sigma Aldrich (Steinheim, Germany).

### Cell isolation, freezing procedure, and thawing of cells

Blood was deposited into BD Vacutainer-EDTA-Tubes (REF 368861) and CPTTM Cell Preparation Tubes (two tubes á 9 ml per patient). Deposited heparinized blood from the examined subjects was used as the source of peripheral blood mononuclear cells (PBMCs). After centrifugation at 430×g for 20 minutes at 21°C, the PBMC fraction was collected and washed twice in 50 ml of sterile PBS (phosphate buffered sodium chloride). The cells were suspended in 1500 µl of sterile fetal calf serum (FCS). In three sterile cryo tubes, 400 µl of sterile FCS were prepared and 500 µl of cell solution were added to each of them followed by gradually adding 100 µl of DMSO from Applichem (Darmstadt, Germany) and swinging the closed tube slowly back and forth before freezing it immediately in a Nalgene freezing box in a −80°C refrigerator. Cells were transferred into liquid nitrogen for long-term storage during the following month.

The thawing of the cells was performed according to an established protocol. The cells were counted and their viability was checked using trypan blue (Sigma Aldrich, Steinheim, Germany) exclusion. Cells were thawed and suspended to the desired concentration in cell culture medium, consisting of RPMI enriched with 10% FCS.

### Measurement of IL-8 and IL-1β release from PBMCs

The ELISpot protocol to examine the release of IL-8 and IL-1β by PBMCs was based on the work of Smedmann et al., 2009 [35]. As the stimulation by LPS varies depending on the charge, we performed preliminary tests with healthy cells and found 200 ng/ml to be the concentration that lies completely in the linear range under the used culture conditions. According to the manufacturer's specification the LPS contained less than 1% protein and less than 1% RNA. Prior to coating, each of the PVDF (polyvinylidene difluoride) membrane plates (MSIPS4510 from Millipore) were pre-wetted with 50 µl 35% ethanol/well for 2 minutes and washed five times with 200 µl sterile H_2_O/well. Capture antibodies were diluted in sterile PBS to 7.5 µg/ml of which 100 µl was added to each well of the membrane plate. After incubation overnight at +4°C, the coated wells were washed five times with 200 µl sterile PBS per well followed by blocking the membrane for 30 minutes with 200 µl/well of cell culture medium (RPMI +10% FCS). The medium was then removed and 100 µl/well of the same culture medium with and without stimulant (concentrations: *E. coli* and *Porphyromonas gingivalis* LPS: 200 ng/ml; CWPS: 200 ng/ml; prefabricated solution) were added followed by the addition of 100 µl/well of cell solution (3500 cells per well). We performed double determination. Plates were transferred to a 5% CO_2_-incubator and incubated for 5 hours (for IL-8) and 21 hours (for IL-1β). After incubation the cells were removed by washing five times with 200 µl/well of sterile PBS using a multichannel pipette (Eppendorf). The biotinylated antibodies were diluted in PBS with 0.5% FCS to the concentration of 1 µg/ml and 100 µl were added per well. After incubation for 2 hours at room temperature in the case of IL-1β or overnight at +4°C in the case of IL-8, plates were washed as described above and developed as described in Smedmann et al.

Reading and counting of spots were performed with the AID Spot Reader. We divided the number of spots formed in wells without antigen-stimulation by the number of spots in wells with antigen-stimulation to calculate the stimulation-index (SI) for each stimulant.
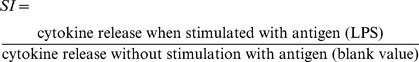



### Flow cytometry diagnostics

#### CVID panel

Peripheral B-cell phenotyping was conducted using flow cytometry. 20 µl of normal serum mix consisting of mouse and goat serum (Dako) were pipetted in each FACS vial with the cell suspension, mixed well, and incubated for 10 minutes at room temperature in the dark. Subsequently, 39.5 µl of an antibody mix made of IgD FITC, CD21 PE, CD5 Per-Cy5.5, CD38 PE-Cy7, IgM APC, CD27 APC-H7, CD19 AmCyan (BD Biosciences, San Jose, California, USA), and CD24 Pacific Blue (EXBIO Praha, Vestec u Prahy, Czech Republic) were pipetted into the vials and mixed well. After another 15 minutes of incubation as described above, lysis buffer (BD Biosciences, San Jose, California USA) was added and the vials were incubated for another 10 minutes. After centrifugation at 250×g for 5 minutes, the supernatant was poured off and cells were suspended in 3 ml of PBS and mixed. After another round of washing the supernatant was discarded and the cell pellet was loosened and fixed by adding 250 µl of PBS with 1% formaldehyde. After fixation, measurement was carried out using a FACS Canto II and analyses were performed with BD Facs Diva.

#### Release of intracellular cytokines in T-helper (Th) cells

After thawing the PBMCs as described above, lymphocytes were stimulated by adding calcium ions, 10 ng/ml phorbol-12-myristate-13-acetate (PMA) (Sigma Aldrich, Steinhein, Germany) and ionomycin (1 µM) (Calbiochem, Merck, Darmstadt, Germany) to activate protein kinase C. To interrupt intracellular protein transport, monensin (2.5 µM) (Sigma Aldrich, Steinheim, Germany) was added. After 5 hours of incubation at 37°C in CO_2_-atmosphere, cells were washed with PBS +10% FCS and fixated for 10 minutes at 4°C. Subsequently, cells were permeabilized and visualization of cell type and cytokine production was conducted simultaneously using CD3 (BD Biosciences, San Jose, California USA), CD4-APC, and CD8-PC5 (IMMUNOTECH SAS, Marseille, France) for visualizing different T-cell-subsets. ALEXA Fluor 647 Mouse anti-Human IL-17A (BD Biosciences, San Diego, California USA), IOTest Anti-IFNγ-PE, and IOTest Anti-IL4-PE (Immunotech, Marseille, France) were used as antibodies to detect the released cytokines. As the percentage of IFN-γ producing T-cells represents the Th1 fraction and IL-4 is a typical Th2 cytokine, we divided the one percentage by the other to illustrate the ratio of Th1 and Th2 cells.

### Microbiological diagnostics

Probes were taken from the four teeth with the highest values of PD and CAL, if possible each from another quadrant. One sterile paper point (ISO 50) was used per site and kept in situ for 10 seconds. All four paper points were immersed in an Eppendorf tube with 1 ml Brain Heart Infusion (BHI) Bouillon (Oxoid, Basingstoke, Hants, UK). The pooled probes were vortexed for 30 seconds and 10-fold serially diluted up to 10^−5^ in thioglycolate broth. For cultivation 100 µl of broth dilution (10^−1^ to 10^−5^) were plated on Columbia blood agar (Oxoid, Basingstoke, Hants, UK) supplemented with or without gentamycin (100 mg) (Oxoid, Basingstoke, Hants, UK). Also, 100 µl aliquots (10^−1^ to 10^−3^) were cultivated on trypticase soy serum bacitracin vancomycin (TSBV) agar (Oxoid, Basingstoke, Hants, UK) for the selective isolation and counting of *A. actinomycetemcomitans*. Columbia plates were incubated anaerobically at 37°C for 7 days and TSBV plates at 37°C in 10% CO_2_ atmosphere for 3 days. Facultative anaerobic strains were not further included in the analysis while anaerobic strains were identified based on Gram stain, colony morphology, production of catalase, An-Ident disc pattern (Oxoid, Basingstoke, Hants, UK), and biochemical tests using the Rapid ID 32A system (BioMerieux, Lyon, France). *A. actinomycetemcomitans* was identified by its typical colony morphology (star-like inner structure) and the production of catalase.

Strains which could not be identified were frozen at −80°C for further analysis.

Frozen samples were thawed at room temperature. Subsequently, the templates were generated and analyzed using the DNeasy Blood and Tissue Kit (Qiagen, Hilden, Germany). 2.0 µl template was mixed with 48.0 µl mastermix (consisting of 36.75 µl distilled water, 5.0 µl buffer, 3.0 µl MgCl_2_, 1.0 µl dNTPs, 1.0 µl BAK, 1.0 µl PC3mod, and 0.25 Taq polymerase). DNA amplification was done with the T-gradient (PCR-blog). 10.0 µl of the thus generated PCR product were plated on an agarose gel to evaluate PCR success. If the characteristic pattern was identified, the product was cleaned using an Invisorb Spin PCRapid Kit (Invitek, Berlin, Germany) according to manufacturer's instructions. The next step of sequencing required production of a mastermix of 8.0 µl distilled water, 4.0 µl marked dNTPs (Interdisciplinary Center for Clinical Research of the Medical Faculty, Leipzig) and 1.0 µl BAK (Biometra, Göttingen, Germany) as a primer. Sequencing was done in the thermocycler using program 3. 2.0 µl of natrium-acetate and 60.0 µl of 96% ethanol were added to the product and incubated for 15 minutes at room temperature followed by another 15 minutes of centrifugation at 1371×g. Subsequently, ethanol was removed carefully and 100.0 µl of 70% ethanol was added and centrifuged as described above. After removing the ethanol the product was dried at room temperature and given to the Interdisciplinary Center for Clinical Research of the Medical Faculty, Leipzig, for further analysis. Identification was based on the 16S ribosome DNA genes [7] and with the aid of the BLAST database (http://blast.ncbi.nlm.nih.gov.Blast.cgi).

Four individuals were not microbiologically characterized because of internal hospital processes/sample handling.

### Statistical analysis

Microsoft Excel 2010 was used for organizing the data. Statistical analyses and graphical display were conducted with SPSS Statistics 14.0 (SPSS Inc.). This program always performs an analysis whether the data follow a Gaussian distribution. Nearly all results did not follow a Gaussian distribution. Therefore, we decided to perform non-parametric analyses for all measurement groups, even in the few ones that did show a Gaussian distribution, because non-parametric tests always deliver accurate results.

We used the Kruskal-Wallis test for analyzing differences between different groups in stimulation indices for IL-8 and IL-1β as determined by ELISpot Assay. Bonferroni adaption was performed and differences were considered to be significant for p<0.008.

Microbiological findings were analyzed using the Chi-Square-test and the Mann-Whitney-U-test. After the Bonferroni correction was applied, p<0.001 was considered significant.

Associations between microbiological and immunological data were investigated with the Mann-Whitney-U-test and the Kruskal-Wallis-Test. Associations with p<0.05 were considered significant and are shown in this article.

## Results

As previously defined in the inclusion criteria, patients with aggressive periodontitis showed increased clinical attachment levels (median 3.4 mm) in accordance with advanced radiographic bone loss and pocket probing depths (median 3.2 mm) as displayed in Table 1. All included subjects were non-smokers and none of them reported any of the medical conditions leading to exclusion from the study. Patients suffering from moderate chronic periodontitis revealed worse oral hygiene represented by higher API values (median 36%) compared to patients with aggressive periodontitis (median 25%) and healthy subjects (median 12.5%). On the other hand, clinical signs of inflammation were much more pronounced in patients with aggressive periodontitis as shown by higher bleeding on probing (BOP) (Table 1).

**Table 1:** Mean Clinical Parameters of Study Patients.

		Aggressive Periodontitis	Chronic Periodontitis	Healthy Controls
		(n = 15)	(n = 11)	(n = 14)
		8 ♀, 7 ♂	5 ♀, 6 ♂	11 ♀, 3 ♂
**Age [years]**	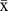 ±σ	32.1±7.1	45.2±8.0	24.9±1.6
	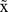	31.0	46.0	25.0
**IPI [%]**	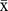 ±σ	30.7±15.2	35.6±16.4	12.1±5.5
	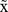	25.0	36.0	12.5
**BOP [%]**	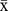 ±σ	30.1±20.1	24.8±14.4	13.6±8.9
	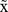	24.6	17.5	17.0
**CAL [mm]**	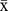 ±σ	3.4±1.0	2.2±0.8	0.4±0.4
	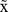	3.4	2.2	0.3
**PD [mm]**	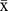 ±σ	3.3±1.0	2.7±0.7	2.0±0.3
	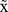	3.1	2.5	2.1
**PD (max) [mm]**	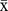 ±σ	7.7±2.7	5.3±1.4	3.6±0.6
	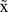	7.0	5.0	4.0
**Bone loss [%]**	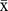 ±σ	66.7±9.8	13.2±4.6	0.0±0.0
	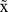	70.0	15.0	0.0

IPI: interproximal plaque index; PD: probing depth; CAL: clinical attachment level; BOP: bleeding on probing; bone loss: maximum of radiographic bone loss.

### Stimulation index for IL-1β significantly elevated in CP group

**Figure 1:**
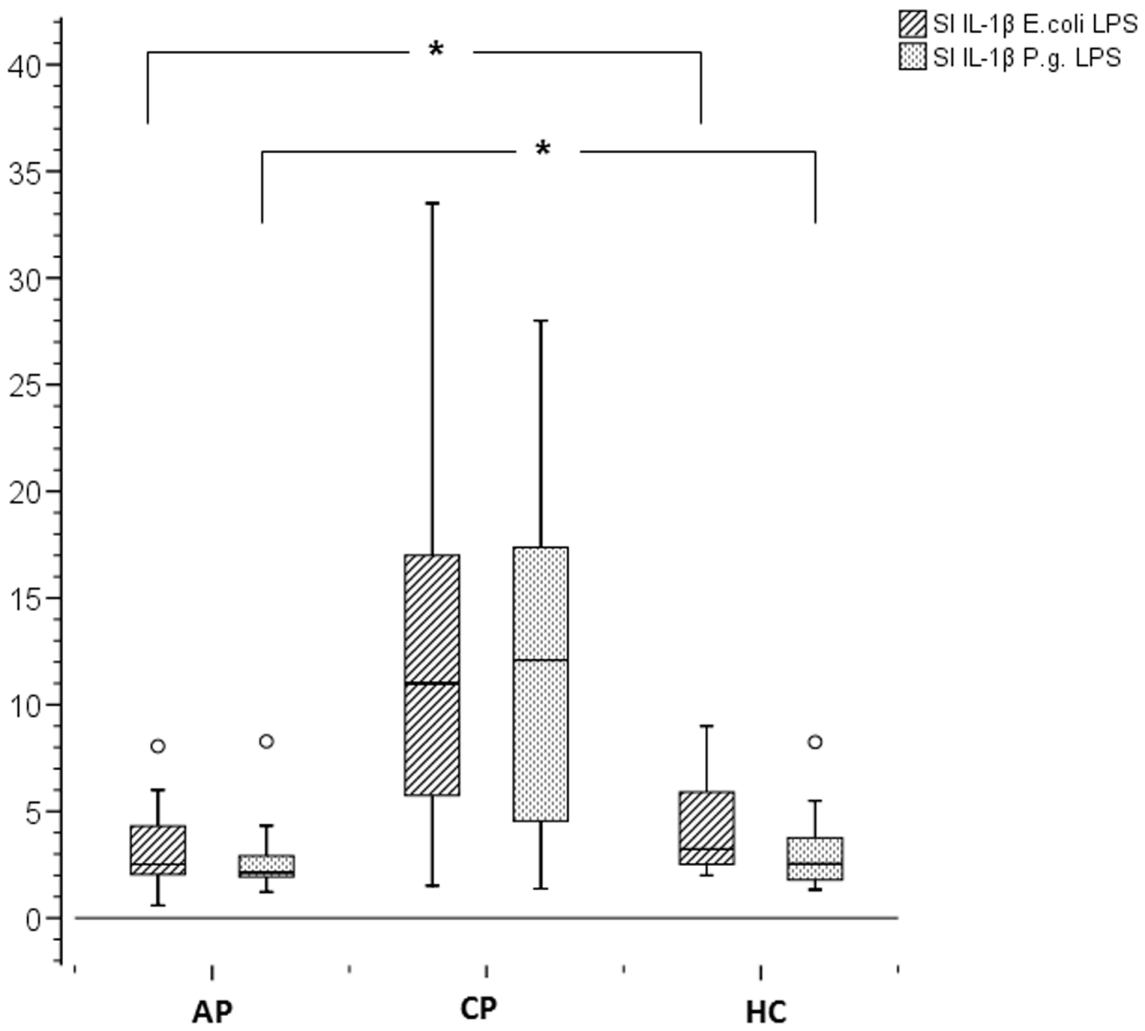
Stimulation Index for IL-1β. PBMCs (3500 cells/well) were stimulated for 20 hours with different forms of LPS (200 ng/ml). Stimulations were run in duplicate. Boxplots represent minimum, first quartile, median, third quartile, and maximum. Differences between the groups were considered to be significant for p<0.008 (Bonferroni adjustment).

In the production of IL-1β by PBMCs, we found no difference between the healthy control (HC) and aggressive periodontitis (AP) groups, as is shown in Figure 1 (grouped boxplots for stimulation index). By contrast, we found significantly elevated stimulation indices in the chronic periodontitis (CP) group when PBMCs were stimulated by both *P. gingivalis* LPS and *E. coli* LPS (p = 0.002). Regarding the release of IL-8 we could not find any differences between the groups (no figure shown).

### Highest percentage of memory B-cells without class switch in CP group

The results of the CVID-panel revealed that no immunodeficiency was present in any of the examined subjects. Numbers of B-cells, naïve and transitional B-cells, memory B-cells, and switched memory B-cells were within the reference range defined by Warnatz et al. [36].

**Figure 2:**
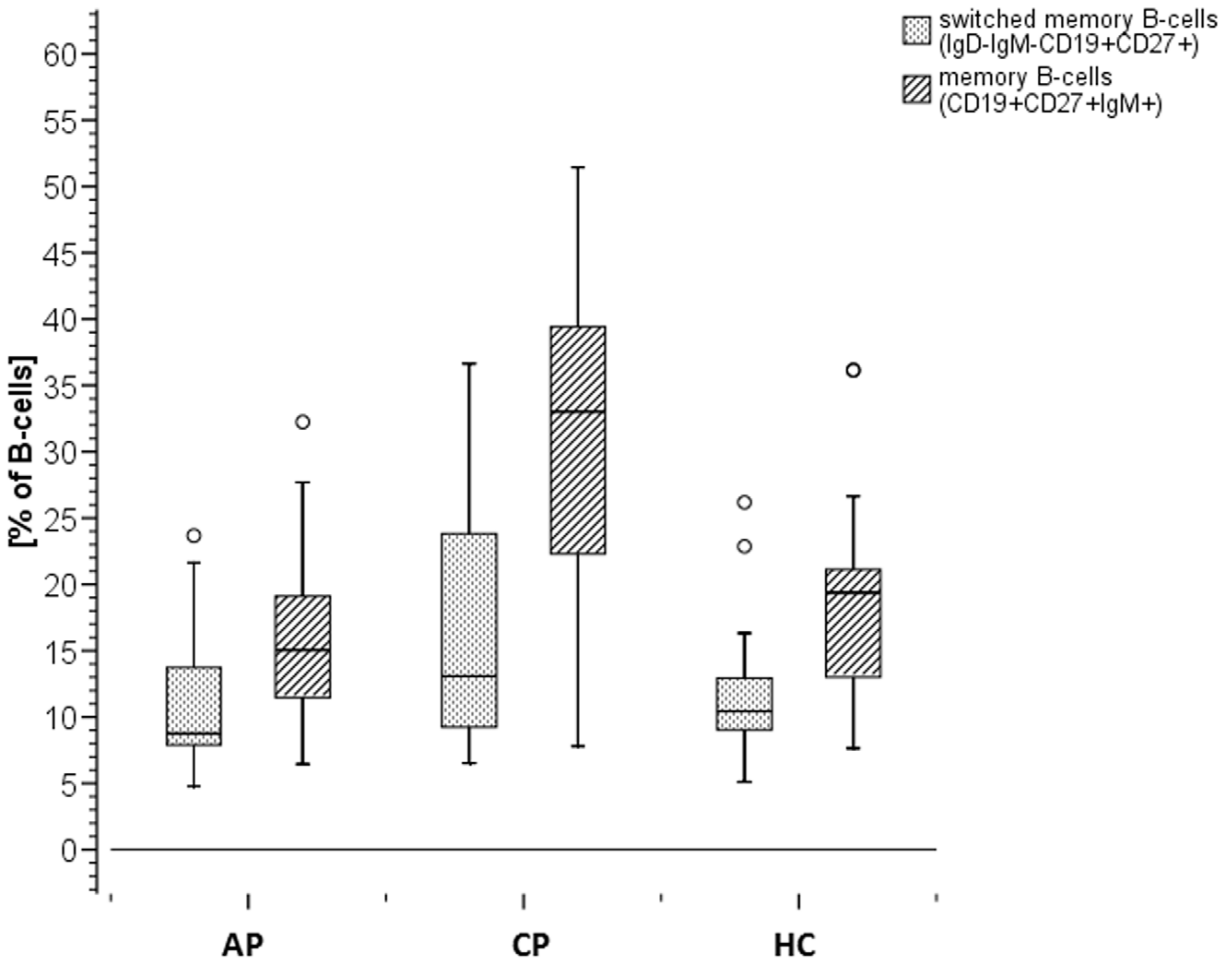
Percentage of Memory B-cells. Memory B-cells as percentage of CD19^+^ B-cells measured by flow cytometry. Explorative analysis revealed a higher level of memory B-cells in patients with moderate chronic periodontitis (median of 33.03%) compared with healthy subjects and patients with aggressive periodontitis (median of 19.39%/15.06%). With a median of 19.39% for all subject groups, an increase in memory B-cells in patients with moderate chronic periodontitis is statistically significant (p = 0.01).

Differences between the three groups were found in the percentage of memory B-cells without class switch as shown in Figure 2 with the highest median value in the CP group (33.03%) compared to the AP (15.06%) and HC (19.39) groups. This difference between moderate chronic periodontitis and the other two groups is significant (p = 0.01).

### Intracellular cytokines showed a slightly reduced Th1 response in AP

**Figure 3:**
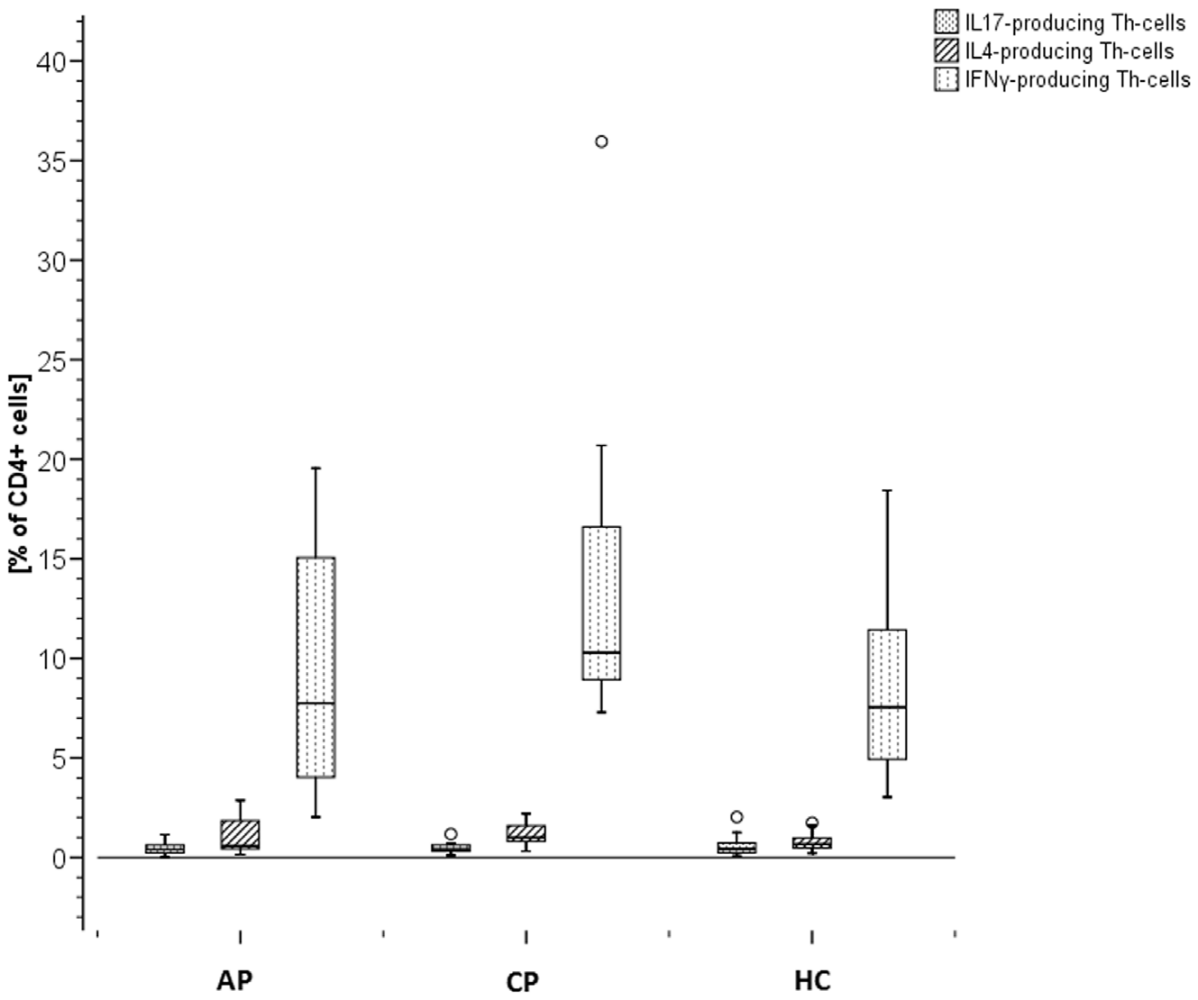
Intracellular Cytokine Release. Production of intracellular cytokines (IL-17, IL-4, IFN-γ) in T-helper cells (Th-cells). Measured by flow cytometry after stimulating CD4+ T-cells with PMA (10 ng/ml).

As shown in Figure 3, regarding IL-4 and IFN-γ, a slightly higher intracellular cytokine secretion was found in PBMCs of subjects with CP with a median of 1.00% of the cells secreting IL-4 and 10.29% of the cells secreting IFN-γ compared to HC (0.67% and 7.55%) and AP (0.58% and 7.74%). Therefore, Th1/Th2-ratios showed a slightly reduced Th1 response in AP patients defined by a lower Th1/Th2 ratio (8.59) compared to healthy controls (10.93) and CP patients (11.83). No differences in PBMC-release of IL-17 when stimulated by PMA between the three groups were found. In each group the percentage of cells secreting IFN-γ was found to be higher compared to cells secreting IL-4 and IL-17.

### Microbiological findings in relation to clinical diagnosis

All patients with chronic periodontitis, 12 of the 15 patients with aggressive periodontitis and 13 of the 14 healthy subjects were microbiologically examined. We identified 236 anaerobic isolates (186 strains from patients, 50 from healthy subjects). 51 different anaerobic species could be identified, with a mean of approximately 8 species per periodontitis patient and almost 4 species per healthy control subject. The detection frequencies of anaerobic bacteria are listed in Table 2. Subjects in whom a given species was detected were considered to be colonized by that species.

**Table 2:** Carriers of Individual Cultivated Species among Different Subject Groups.

Bacterial Species	colonized AP (n_total_ = 12)		colonized CP (n_total_ = 11)		colonized HC (n_total_ = 13)		p-value
	n	%	n	%	n	%	
*Prevotella intermedia* *	9	75.00	9	81.82	5	38.46	0.046
*Fusobacterium nucleatum*	7	58.33	8	72.73	5	38.46	0.355
*Prevotella oralis* *	7	58.33	6	54.55	1	7.69	0.013
Prevotella denticola **	7	58.33	1	9.09	0	0	0.001
*Veillonella species*	7	58.33	2	18.18	5	38.46	0.116
*Actinomyces meyeri*	5	47.92	2	18.18	4	30.78	0.682
*Prevotella loeschii*	5	47.92	4	36.36	3	23.08	0.618
*Micromonas micros* *	5	47.92	2	18.18	0	0	0.042
*Anaerococcus prevotii* *	4	33.33	0	0.00	0	0	0.011
*Fusobacterium necrogenes*	3	25.00	1	9.09	1	7.69	0.377
*Bacteroides ureolyticus*	3	25.00	3	27.27	1	7.69	0.449
*Prevotella buccae*	3	25.00	6	54.55	3	23.08	0.295
*Selenomonas* spp.	3	25.00	2	18.18	0	0	0.037
*Eggerthella lenta*	2	16.67	1	9.09	3	23.08	0.614
*Capnocytophaga spp.*	2	16.67	3	27.27	6	46.15	0.258
*Prevotella melaninogenica*	2	16.67	0	0.00	2	15.38	0.349
*Gemella morbillorum*	2	16.67	5	45.45	2	15.38	0.243
*Peptostreptococcus anaerobicus*	2	16.67	1	9.09	0	0	0.322
*Eubacterium limosum*	2	16.67	0	0.00	0	0	0.117
*Clostridium histolyticum*	2	16.67	0	0.00	0	0	0.117
*Campylobacter rectus*	2	16.67	0	0.00	0	0	0.117
*Clostridium beijerium*	2	16.67	1	9.09	0	0	0.307
*Actinomyces israelii*	1	8.33	1	9.09	2	15.38	0.809
*Actinomyces odontolyticus*	1	8.33	1	9.09	1	7.46	0.998
*Actinomyces naeslundii*	1	8.33	0	0.00	0	0	0.353
*Fusobacterium varium*	1	8.33	1	9.09	0	0	0.573
*Fusobacterium necrophorum*	1	8.33	0	0.00	1	7.69	0.609
*Clostridium bifermentans*	1	8.33	0	0.00	0	0	0.353
*Prevotella buccalis*	1	8.33	1	9.09	0	0	0.573
*Propionibacterium acnes*	1	8.33	1	9.09	1	7.69	0.998
*Propionibacterium propionicus*	1	8.33	0	0.00	0	0	0.353
*Bacteroides merdae*	1	8.33	1	9.09	0	0	0.573
*Eubacterium yurii*	1	8.33	0	0.00	0	0	0.353
*Actinomyces viscosus*	0	0.00	1	9.09	0	0	0.353
*Actinomyces prevotii*	0	0.00	1	9.09	0	0	0.011
*Prevotella bivia*	0	0.00	0	0.00	1	7.69	0.397
*Prevotella disiens* *	0	0.00	2	18.18	0	0	0.037
*Prevotella tannerae*	0	0.00	1	9.09	0	0	0.353
*Prevotella nigrescens*	0	0.00	0	0.00	1	7.69	0.397
*Eubacterium brachy*	0	0.00	1	9.09	0	0	0.353
*Finegoldia magna*	0	0.00	2	18.18	0	0	0.117
*Porphyromonas gingivalis* *	0	0.00	3	27.27	0	0	0.011
*Porphyromonas asaccharolytica*	0	0.00	2	18.18	0	0	0.117
*Porphyromonas endodontalis*	0	0.00	1	9.09	0	0	0.353
*Clostridium clostridioforme*	0	0.00	1	9.09	0	0	0.117
*Clostridium fallax*	0	0.00	1	9.09	0	0	0.117
*Clostridium tyrobutyricum*	0	0.00	1	9.09	0	0	0.117
*Clostridium* spp.	0	0.00	0	0.00	1	7.69	0.397
*Clostridium sordellii*	0	0.00	1	9.09	0	0	0.353
*Clostridium innocuum*	0	0.00	1	9.09	1	7.69	0.609
*Clostridium sporogenes*	0	0.00	1	9.09	1	7.69	0.609

* <0.05; ** <0.001.

Only *Prevotella denticola* was significantly associated with AP and CAL (p = 0.001). All other associations were not statistically significant. *P. intermedia* and *F. nucleatum* were also found in healthy subjects but in lower quantities. We did not detect *A. actinomycetemcomitans* in any of our samples.

### Microbiology in relation to immunological findings

We examined associations between immunological and microbiological findings in an exploratory way to find possible links and generate topics for further research. Findings with p<0.05 are shown in Table 3, irrespective of periodontal diagnosis. For *Prevotella denticola*, which was shown to be associated with aggressive periodontitis, we could not find any association with any of the examined immunological parameters. *P. gingivalis*, which was found only in the CP group, showed an association to higher stimulability of PBMC-release of IL-1β and IL-8. Furthermore, this strain could be associated with the percentage of IFN-γ-releasing T-helper cells, which leads to a higher IFN-γ/IL-4 ratio in relation to intracellular cytokine release in T-helper cells. Additionally, we found that patients who harbor *P. g*. in their subgingival microflora have a shift in CD4/CD8 ratio towards CD8.

**Table 3:** Associations between Immunological and Microbiological Findings.

Bacterial Species	Immunological Finding	Association	p	Interpretation
*P. gingivalis* (n = 3)	SI IL-1β *E.coli* LPS	positive	0.019	1
	SI IL-8 *E.coli* LPS	positive	0.029	1
	SI IL-8 *P.g.* LPS	positive	0.015	1
	CD4/CD8 ratio	negative	0.023	1
	IFN-γ producing T-helper cells	positive	0.023	1
	IFN-γ/IL-4 ratio	positive	0.015	1
*F. nucleatum* (n = 20)	B-cells	negative	0.030	2
	naïve and transitional B-cells	negative	0.050	2
	switched memory B-cells	positive	0.014	2
	memory B-cells	positive	0.018	2
*A. prevotii* (n = 4)	SI IL-1β *E.coli* LPS	negative	0.007	3
*A. meyeri* (n = 12)	B-cells	negative	0.049	4
*P. disiens* (n = 2)	SI IL-1β *P.g.* LPS	positive	0.029	5
	IFN-γ/IL-4 ratio	positive	0.029	5
*C. rectus* (n = 2)	SI IL-1β *E.coli* LPS	negative	0.038	3
	IFN-γ producing T-helper cells	negative	0.019	3
*E. limosum* (n = 2)	naïve and transitional B-cells	positive	0.013	2
	B-cells	positive	0.013	2
*Finegoldia magna* (n = 2)	B-cells	positive	0.013	6
	IFN-γ/IL-4 ratio	negative	0.019	6
	SI IL-8 *E.coli* LPS	negative	0.038	6
	IL-4 producing T-helper cells	positive	0.038	6
*P. oralis* (n = 14)	switched memory B-cells	negative	0.035	2
	IFN-γ/IL-4 ratio	negative	0.035	2
*P. buccalis* (n = 2)	CD4/CD8 ratio	positive	0.003	

n: positive identifications from the 46 microbiologically characterized individuals; SI: stimulation index;

1 activation of Th1-response;

2 activation of B-cell-maturation;

3 negative regulation of innate immunity;

4 no B-cell stimulation;

5 positive regulation of innate immunity;

6 activation of humoral immunity (Th2).

For patients harboring *Prevotella disiens* in their subgingival flora, a higher stimulation index (SI) was found for the release of IL-1β when cells were stimulated with *P. g.* LPS, and a higher IFN-γ/IL-4 ratio was also found. Furthermore, *Prevotella intermedia* harboring patients showed a lower percentage of switched memory B-cells.

In summary, as shown in Table 3, we found nine associations for T- and B-cells, which are representative for adaptive immunity, with microbiological strains. Five associations were found for SI of IL-1β release and two associations for SI of IL-8 release, both representing innate immunity.

## Discussion

In this study we included patients suffering from aggressive periodontitis as well as patients with chronic periodontitis and healthy subjects. We collected clinical data and isolated the PBMCs from peripheral blood of each subject in order to examine the reaction to stimulation with standardized extracts of bacterial toxins (LPS from *E. coli* and *P. gingivalis*). Furthermore, we investigated T-cells and B-cells as well as the microbiological composition of patients' subgingival microbiota. Although there are differences in age existing between the three groups according to the inclusion criteria used in this study, they are not large enough to expect them to be the reason for the immunological differences we have found. We have no reason to suspect the unequal proportion of men and women between the groups influenced the study results. A systematic review by Shiau and Reynolds found a greater risk for destructive periodontal disease in men than in women, but without a higher risk for more rapid periodontal destruction [37]. As we performed analyses with the PBMCs in vitro an influence of gender mediated by hormones, biofilm or other factors is not to be expected. No other differences concerning potential risk factors for periodontal diseases exist between the groups.

Generally, immunological investigations of regulatory mechanisms in the pathogenesis of periodontitis can be performed with different types of cells from different origins in the organism. It is important to note that the local immune response is not determined only by PBMCs, but these cells represent the systemic immune status.

Recent studies focused on innate immunity in periodontitis pathogenesis. Production of IL-1 upon bacterial stimulation has been of special interest [38]. As many authors found that most patients suffering from each form of periodontitis have a mixed colonization of subgingival microbiota, we did not use a specific bacterial extract for stimulating PBMCs but two different standardized LPSs (from *E. coli* and *P. gingivalis*). *E. coli* LPS was used as it has a similar structure to LPS of the periodontal pathogen *A. actinomycetemcomitans*, and is a TLR-4 agonist [39]. Ultrapure LPS from *Porphyromonas gingivalis* is an agonist of TLR-4 as well. The TLR-2 activity of *P. gingivalis* LPS is ascribed to a contaminant lipoprotein [40] which is not present in ultrapure LPS because of the enzymatic treatment. Therefore, an activation of the TLR-2 cannot be assumed. As shown in Figure 1, for both LPSs, a significantly higher stimulation of IL-1β production was found in patients with moderate chronic periodontitis compared to the other groups (p = 0.002), indicating a higher activation of innate immunity in moderate chronic periodontitis. Many studies have investigated IL-1β in gingival tissues and sulcus fluid. They showed a positive association between the local expression of IL-1β and severity of periodontal disease [41], [42]. On the other hand, plasma levels of all the interleukins studied (IL-1β, IL-6, IL-11) were not significantly different between study groups (chronic periodontitis, generalized aggressive periodontitis, gingivitis, healthy subjects) [42]. Our results show that IL-1β release by PBMCs after stimulation with LPS in patients with AP is approximately identical to healthy controls, but increased in patients with CP. This finding does not correlate with the fact that patients with CP show a milder disease progression, as IL-1β is a proinflammatory cytokine. In contrast, other authors hypothesized that a reduction in levels of IL-1β could attenuate the host's ability to fight an infection [43]. A possible explanation could be the turnover of neutrophils in gingival tissue, which seems to be reduced in patients with aggressive periodontitis because of less TNF-α [44]. In patients with moderate chronic periodontitis, the immunological homeostasis in relation to neutrophil turnover seems to be sustained, maybe because of IL-1β, which compensates for TNF-α, resulting in limited inflammation.

We did not find differences in the release of IL-8 by PBMCs when stimulated with LPS between the different groups. IL-8 is an important pro-inflammatory chemokine which is regulated by an activator-protein and/or by NF-κB-mediated transcriptional activity and some other stimuli [45]. Goncalves et al. found a higher LPS-induced release of IL-8 in healthy subjects' PBMCs compared to patients suffering from chronic periodontitis [46]. In contrast to Goncalves, Dias et al. found higher plasma concentrations of IL-8 in patients with severe chronic periodontitis compared to healthy controls [47]. This indicates that local processes do not reflect systemic immune regulation one-to-one.

The final immune response takes place on the local level and is crucial for the regulation of the microbiota by stimulating microenvironmental changes limiting the increase of periodontal pathogens and helping protective bacteria to predominate or by permitting the establishment of a more pathogenic microbial ecology [48]. Most of the local immunological factors belong to the innate immune system with monocytes (CD14+) representing one of the principal peripheral mononuclear cells. When monocytes migrate into the periodontal tissue they convert to macrophages. However, previous studies suggested that a bacteremia that is caused by periodontitis [49] might induce cytokines to change the immune cell function (e.g. developing CD14+ and CD16+ monocytes [50]). On the other hand, these changes might also be triggered by cytokines released from periodontal lesions. Therefore, it was of special interest for us not only to determine associations between microbiological and clinical but also immunological findings. We showed predominance of known periodontal pathogens in the periodontitis groups (Table 2) and found differences in microbial colonization between healthy and diseased sites in quantity as well as in quality as other authors have described before [51]. In this study, a higher quantity of Gram-negative anaerobic bacteria was detected in periodontitis patients compared to healthy subjects: *P. gingivalis*, *P. intermedia*, *P. oralis*, *Parvimonas micra*, and *Prevotella denticola* were associated with periodontitis. These findings support previous results of other research groups who correlated *P. gingivalis* and *P. intermedia* with periodontal lesions [51], [52]. As shown by other authors and summarized by Mombelli et al. [53], we also found no differences in the microbiological colonization between patients in the AP and CP groups. Furthermore, we found known periodontopathogenic species: *Veillonella*, *Capnocytophaga*, *F. nucleatum*, and *P. intermedia* in the sulcus of healthy individuals as has been shown before [15], [54].

Our microbiological and immunological findings allowed us to identify links between several periodontal pathogens and adaptive as well as innate immune response (Table 3). These associations should be interpreted cautiously because of multiple testing and limited sample sizes, which is why we regard these results as exploratory. We found an association between *P. gingivalis* and Th1-response which is contradictory to a recent study of Moutsopoulos et al. who were able to show that *P. gingivalis* W83 stimulates myeloid antigen presenting cell (APC) differentiation to Th17-cells in vitro by activating NFκB and thereby inducing IL-1β, IL-6, and IL-12p40 [55]. On the other hand, an older study from 2002, which investigated the influence of *P. gingivalis* LPS on the accumulation of IL-12 and IFN-γ in T-cells, suggested an increase in the production of inflammatory cytokines caused by an activation loop with IL-12 and IFN-γ established by *P. gingivalis* [56]. Thus, this study supports our results. As we indicated above, there remains widespread disagreement in the literature regarding the systemic immunopathology of periodontitis.

Considering B-cell maturation, we found the highest percentage of memory B-cells in the CP group as shown in Figure 2, suggesting a stronger antibody-mediated systemic immune response towards infection than in the other groups. This assumption is based on the fact that IL-1β can induce production of IL-6, which leads to the activation of lymphocytes and their release of immunoglobulin [30]. This results in a higher release of IgG in patients with moderate chronic periodontitis than in the other groups. As a reduced production of IgG could be associated with severity of periodontitis [57], this could be an explanation for the milder progress of illness in patients with moderate chronic periodontitis and an illustration for the interaction between innate and adaptive immunity.

One of the main strengths of this study is that we determined well-defined cohorts with numbers of individuals that were adapted to the incidence of aggressive periodontitis. Furthermore, we collected periodontological, microbiological and functional as well as phenotypic immunological data, which enabled us to characterize the individuals in a comprehensive way. Nevertheless, the number of individuals is relatively low. Therefore, some of the results can only be considered as exploratory and a direct connection between local and systemic immunological factors cannot be assumed. Therefore, these points represent limitations of our study. Further studies should follow-up the immunological and microbiological results described in our study, preferably on the basis of an appropriate number of individuals, to characterize the connection between systemic factors and local factors in the periodontium more precisely.

## Overall Conclusions

The results of this study provide evidence for possible associations between clinical diagnosis, immunological findings, and periodontal pathogens. Considering systemic immune status we found a significantly higher activation of innate and adaptive immunity in patients with moderate chronic periodontitis compared to the other groups, represented by the significantly higher stimulability of PBMCs in IL-1β release and the elevated percentage of memory B-cells compared to the other groups. Furthermore, we were able to show an association between the presence of bacteria typical for periodontal disease and the systemic immune response. The results indicate that in periodontal disease there is some kind of self-limitation of inflammation in patients diagnosed with moderate chronic periodontitis. In such instances, a clearly milder disease progression is observed as compared to patients with aggressive periodontitis.
